# The complexity of TRIM28 contribution to cancer

**DOI:** 10.1186/s12929-017-0374-4

**Published:** 2017-08-29

**Authors:** Patrycja Czerwińska, Sylwia Mazurek, Maciej Wiznerowicz

**Affiliations:** 1 Laboratory of Gene Therapy, Department of Diagnostics and Cancer Immunology, Greater Poland Cancer Centre, 15 Garbary Street, 61-866 Poznan, Poland; 2 Department of Cancer Immunology, Chair of Medical Biotechnology, Poznan University of Medical Sciences, Poznan, Poland; 3 Postgraduate School of Molecular Medicine, Medical University of Warsaw, Warsaw, Poland

**Keywords:** TRIM28, KAP1, Cancer, Transcriptional co-repressor, EMT, Autophagy, Cancer stem cells

## Abstract

Since the first discovery in 1996, the engagement of TRIM28 in distinct aspects of cellular biology has been extensively studied resulting in identification of a complex nature of TRIM28 protein. In this review, we summarize core biological functions of TRIM28 that emerge from TRIM28 multi-domain structure and possessed enzymatic activities. Moreover, we will discuss whether the complexity of TRIM28 engagement in cancer biology makes TRIM28 a possible candidate for targeted anti-cancer therapy. Briefly, we will demonstrate the role of TRIM28 in regulation of target gene transcription, response to DNA damage, downregulation of p53 activity, stimulation of epithelial-to-mesenchymal transition, stemness sustainability, induction of autophagy and regulation of retrotransposition, to provide the answer whether TRIM28 functions as a stimulator or inhibitor of tumorigenesis. To date, number of studies demonstrate significant upregulation of *TRIM28* expression in cancer tissues which correlates with worse overall patient survival, suggesting that TRIM28 supports cancer progression. Here, we present distinct aspects of TRIM28 involvement in regulation of cancer cell homeostasis which collectively imply pro-tumorigenic character of TRIM28. Thorough analyses are further needed to verify whether TRIM28 possess the potential to become a new anti-cancer target.

## Background

**Fig. 1:**
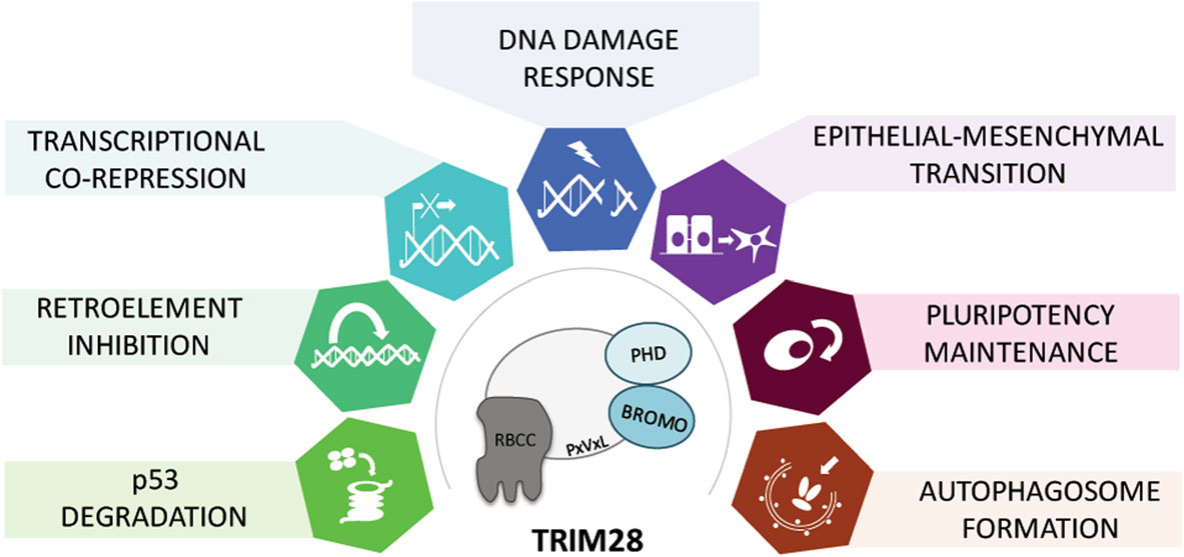
TRIM28 multi-domain protein contributes to the regulation of a variety of intracellular processes. Core biological functions of TRIM28 including inhibition and degradation of p53 tumor suppressor, regulation of retrotransposition, regulation of gene expression through heterochromatization, mediation of DNA damage response, stimulation of EMT, maintenance of stem cell pluripotency and stimulation of autophagophore formation (induction of autophagy) are frequently intercepted by cancer cells to promote the proliferation and acquire resistance to stress inducing agents

This work is focused on highly complex TRIM28 protein which role in the biology of normal and cancer cell has been studied for over 20 years [1–3]. First described as a universal co-factor for a huge family of Krüppel-Associated Box Zinc Finger Protein (KRAB-ZFP) transcription factors [2], TRIM28 is now known to participate in many aspects of cellular biology, either promoting cell proliferation [4] or mediating anti-proliferative activities [5]. Trying to answer the question whether TRIM28 acts as a tumor-inhibiting or tumor-stimulating factor, we delineate the cancer-related roles of TRIM28. Here we demonstrate the involvement of TRIM28 protein in regulation of gene expression through heterochromatin formation, mediation of DNA damage response, inhibition of p53 activity through intrinsic E3 ubiquitin ligase activity, regulation of epithelial to mesenchymal transition (EMT) and maintenance of stem cell pluripotency as well as regulation of autophagy and safeguarding the genome stability through inhibition of retrotransposition (Fig. 1).

## Structure and post-translational modifications of TRIM28 protein

**Fig. 2:**
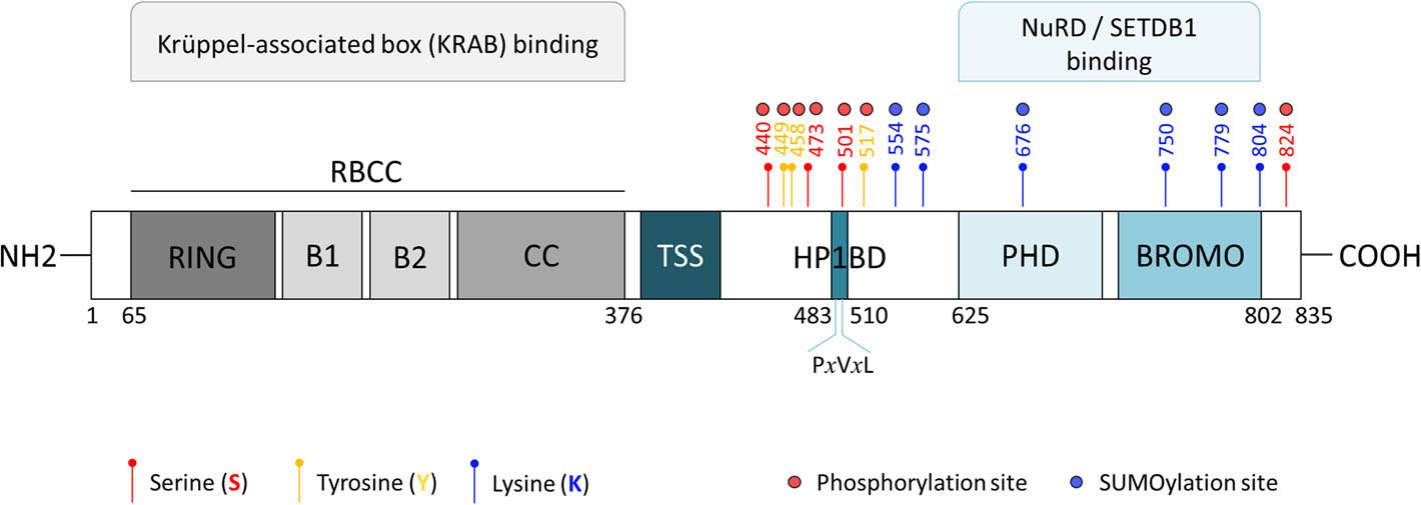
Structure and post-translational modifications of TRIM28 protein. RING – Really Interesting New Gene; zinc finger type domain which contains a C_3_HC_4_ amino acid motif that binds two zinc cations; has an intrinsic E3 Ubiquitin ligase activity; B1, B2 – B-box type 1 and B-box type zinc finger type domains of around 40 amino acid residues in length; CC – Coiled Coil, a structural motif in which 2 alpha-helices are coiled together (like the strands of a rope); RBCC – RING domain followed by B-boxes and CC domain, RBCC domain is responsible for interaction with KRAB domain of KRAB-ZFPs; TSS – TRIM Specific Sequence; HP1BD – HP1 protein binding domain, contains a consensus P*x*V*x*L motif; PHD – Plant Homeodomain, zinc finger type domain which contains a C_4_HC_3_ amino acid motif that binds two zinc cations, has an intrinsic E3 SUMO ligase activity; BROMO – Bromodomain, together with PHD are responsible for NuRD/SETDB1 recruitment and binding

TRIM28 protein, a large multi-domain protein (110 kDa), which is a member of a family of almost 60 human Tripartite motif-containing (TRIM) proteins, is also known as KAP1 (Krüppel-Associated Box (KRAB)-Associated Protein 1) or TIF1-β (Transcriptional Intermediary Factor 1 β) [2, 6]. TRIM28 shares many structural features with three other TRIM proteins, TRIM24 (TIF1α), TRIM33 (TIF1γ), and TRIM66 (TIF1δ), and together constitute a Transcriptional Intermediary Factor 1 (TIF1) family [7, 8]. At the amino (N) terminus, TRIM28 protein contains four conserved structural domains that include a RING (Really Interesting New Gene) finger, two B-boxes, and a leucine zipper coiled-coil region (CC), which are collectively called the RBCC or TRIM domain (Fig. 2) [9, 10]. RING finger domain is a cysteine-rich sequence characterized by the amino acid signature Cys_3_-His-Cys_4_ which binds two zinc cations and together with two B-Boxes and a leucine zipper, is responsible for the interaction with the KRAB domain present in a very large set of the KRAB- Zinc Finger (KRAB-ZFP) transcription factors [2, 9, 11].

The central part of the TRIM28 protein contains the P*x*V*x*L pentapeptide region that mediates interaction with Heterochromatin Protein 1 (HP1). In addition, TRIM28 protein also shares with other TIF1 proteins the central TIF1 signature sequence (TSS) consisting of a 25-amino acid tryptophan- and phenylalanine-rich sequence [12].

At the carboxyl (C) terminus of TRIM28, the plant homeodomain (PHD) finger and the Bromodomain are located, recruiting components of the Nucleosome Remodeling Deacetylase (NuRD), histone deacetylase complex and the histone H3 lysine 9 (H3K9)-specific methyltransferase SETDB1 in order to condense chromatin. The PHD finger, as well as the RING domain, is a cysteine/histidine rich structure and contains a consensus Cys_4_-His-Cys_3_ that spans 50–80 residues. The PHD, Bromodomain, and P*x*V*x*L domain are thought to cooperatively form condensed heterochromatin characterized by the low histone acetylation, high tri-methylation of H3K9 (H3K9me3), and high HP1 binding [13, 14]. This co-repressive activity of TRIM28, including the recruitment of SETDB1 and the NuRD complex and interaction with HP1 protein, is dependent on the SUMOylation state of at least 3 lysine residues (K554, K779, and K804), with TRIM28 itself functioning as an intramolecular E3 SUMO ligase. The PHD domain of TRIM28 protein binds to Ubc9 and directs SUMO conjugation of an adjacent Bromodomain, and this SUMOylation is required for KRAB- TRIM28-mediated gene repression [14].

## TRIM28 expression in cancer

**Fig. 3:**
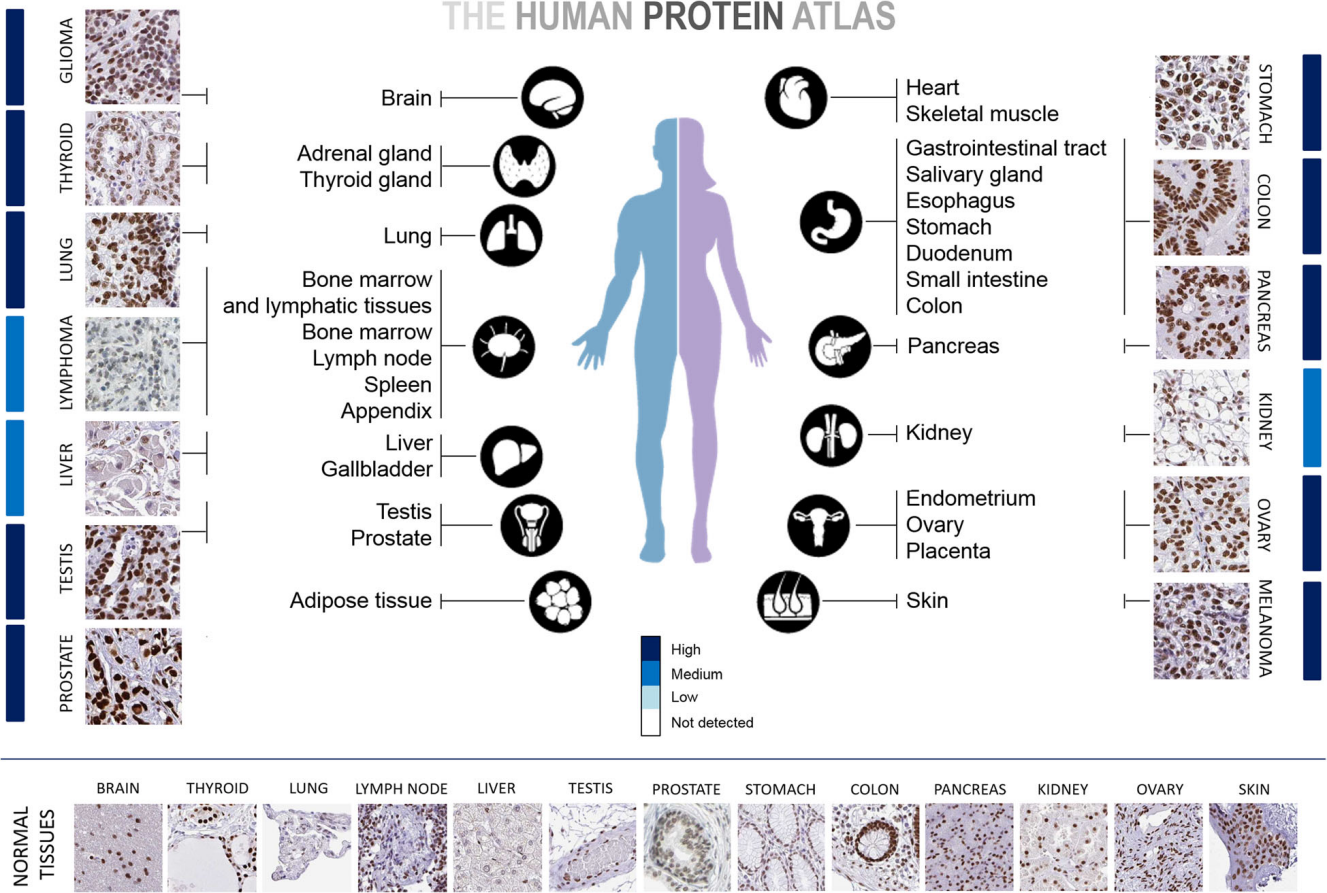
TRIM28 is highly expressed in many types of tumors (The Human Protein Atlas data). Immunohistochemistry was performed with rabbit polyclonal antibody HPA064033 (Sigma Aldrich). Strong nuclear staining is observed in almost all types of cancers presented in The Human Protein Atlas database. Only lymphoma and liver and kidney tumors present medium intensity for TRIM28 staining. Normal tissues showed moderate to strong nuclear positivity with HPA064033 antibody as presented at the bottom, however in most cancer types the presence of TRIM28 in cancer cell nuclei is more pronounced than in adjacent normal tissues

To date, the clinical relevance of TRIM28 in diseases remains elusive. However, several reports revealed positive correlation between the level of *TRIM28* expression and cancer prognosis in specific cancer types. TRIM28 is ubiquitously expressed throughout development, with very high expression in embryonic stem cells [15] and several types of tumors (see below). Higher *TRIM28* gene expression has been linked to prometastatic cervival cancer [16]. Moreover, the upregulation of *TRIM28* gene in tumor tissues has been shown in gastric cancer and is associated with poor prognosis [17]; *TRIM28* gene overexpression was also detected in peripheral blood of gastric cancer patients [18]. The expression level of *TRIM28* was also higher in ovarian cancer samples than in matched non-tumor ovarian tissues and correlated with aggressive clinical features [19]. Furthermore, *TRIM28* high expression was an independent predictor for ovarian cancer patients [20]. *TRIM28* was also highly expressed in glioma when compared to non-glioma controls and its expression was positively correlated with tumor malignancy, and associated with poor overall survival (OS) and progression-free survival (PFS) [21]. Moreover, recent studies discovered TRIM28 as a possible tumor-class predictive biomarker, used to distinguish glioblastomas from lower grade gliomas, and from reference samples with a unique nanobody-based anti-proteome approach [22]. Furthermore, both the mRNA and protein level of TRIM28 were significantly higher in tumor tissues than in adjacent normal tissues in hepatocellular carcinoma patients (HCC). Importantly, the expression of *TRIM28* was closely correlated with tumor size, tumor stage and 5-year overall survival in HCC patients, showing remarkably shorter survival rate in patients with *TRIM28* overexpression [23]. Consequently, TRIM28 was suggested as an independent prognosis factor to predict the survival rate of HCC patient. Previous studies have shown that *TRIM28* was overexpressed in both liver and peritoneal metastases from patients with colorectal adenocarcinoma, melanoma and malignant thyroid tumors [24]. Similarly, analysis of tissue microarrays demonstrated that *TRIM28* level is increased during the clinical progression of nearly 40% of invasive breast carcinomas in situ to metastasis in lymph nodes [4]. Results obtained in our laboratory [25] are in agreement with data published by Addison JB. et al. [4], demonstrating significant upregulation of *TRIM28* gene expression in all four intrinsic breast cancer subtypes and in breast cancer metastases when compared to normal tissue. Moreover, TRIM28 level positively correlates with aggressiveness of breast cancers as reported by Wei C. et al. [26]. Immunohistochemical analysis has revealed higher TRIM28 levels in significant proportions of breast, lung, liver, gastric, and prostate tumors (Fig. 3; for more information see TRIM28 at www.proteinatlas.org), suggesting that TRIM28 upregulation is a common feature of many epithelial cancers [16–21, 23–26].

Summing up, aforementioned studies suggest that higher TRIM28 level is linked to a poor prognosis in certain cancers. However, opposite conclusions were also reported. In early-stage lung cancer higher expression of *TRIM28* gene is associated with better overall survival [5], suggesting that TRIM28 may have also anti-proliferative activity within tumor cells.

Furthermore, liver-specific ablation of *Trim28* in mice increases male-predominant hepatic adenoma, suggesting that TRIM28 protects liver cells from tumorigenic conversion [27]. Indeed, Cassano M. et al. [28] have recently confirmed that Trim28 is a crucial mediator of sexual dimorphism in the liver, tightly regulating the expression of a wide range of bile and steroid metabolism genes. They have demonstrated that hepatocyte-specific *Trim28* knockout mice exhibit alterations of transcriptional dynamics exacerbated by environmental insults such as obesity and ageing, consequently leading to male-restricted development of hepatocellular carcinomas [28]. Therefore, TRIM28 is essential for non-tumor cells to maintain their unchanged phenotype.

## TRIM28 recruitment to the genome and transcriptional co-regulation

**Fig. 4:**
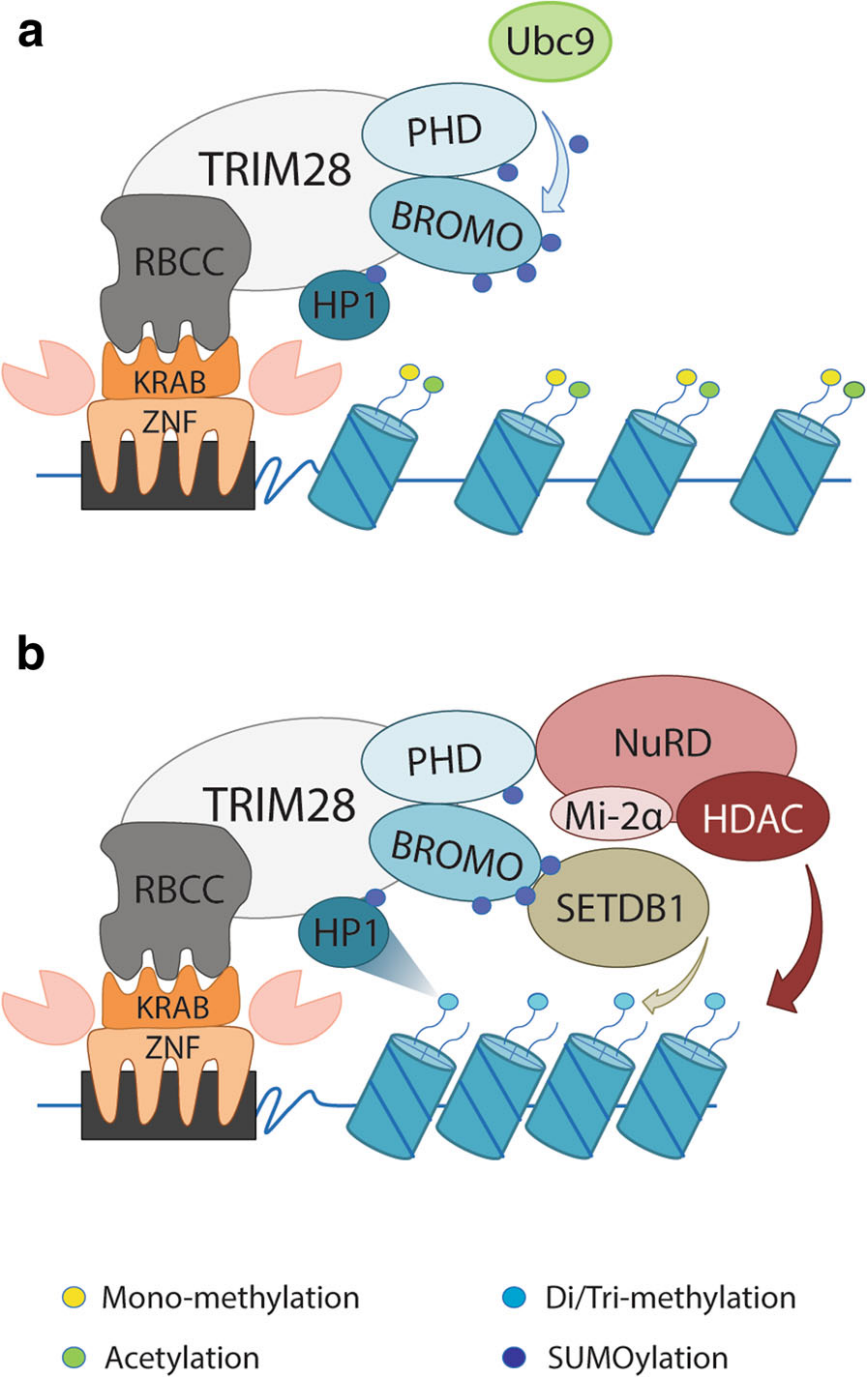
TRIM28 is recruited to the genome by KRAB-ZNF transcription factors to facilitate chromatin compaction-mediated gene repression. a TRIM28 binds to KRAB domain of KRAB-ZNF proteins tethered to specific sequences within the genome using N-terminal RING-B-boxes-Coiled-Coil (RBCC) domain and catalyzes auto-SUMOylation of Bromodomain (utilizing Ubc9 as an E2 SUMO ligase). b SUMOylated TRIM28 recruits SETDB1 and NuRD complex proteins leading to the creation of the H3K9me3 mark on nearby nucleosomes together with deacetylation of histone proteins. HP1 binding further stabilizes the TRIM28-containing complex bound to the KRAB-ZNF

As a transcriptional co-repressor, TRIM28 protein is essential for KRAB-ZNF proteins to unleash their repressive potential. The KRAB-ZNF gene family is specific to tetrapod vertebrates [29] and there are more than 400 human KRAB-ZNF genes encoding transcripts for more than 700 different proteins [30]. This huge family of transcription factors is postulated to regulate diverse processes such as embryonic development, tissue-specific gene expression, and cancer progression [31]. Molecular mechanism of KRAB-ZNF-mediated transcriptional regulation depends on the interaction with chromatin-remodeling factors through the TRIM28 protein (and the formation of transcriptional repressor complex KRAB-ZNF-TRIM28 protein has been mainly studied using artificial assays) [32, 33]. Briefly, KRAB-ZNF proteins bound to specific DNA recognition motifs (transcription factor binding site, TFBS) through their zinc finger domains recruit TRIM28 protein which acts as a scaffold for various heterochromatin-inducing factors. As mentioned previously, this enrollment is dependent upon the specific interaction of the TRIM28 N-terminal RBCC domain with a conserved KRAB repression domain [9–11]. Next, PHD-mediated SUMOylation of bromodomain and resulting recruitment of SETDB1 and NuRD complex proteins lead to the creation of the H3K9me3 mark on nearby nucleosomes together with deacetylation of histone proteins (Fig. 4) [13, 14]. Further HP1 protein binding to TRIM28 at the P*x*V*x*L motif and to the H3K9me3 mark, subsequently stabilize the TRIM28-containing complex bound to the KRAB-ZNF [34]. It should be noted that neither TRIM28 nor any of the aforementioned proteins recruited by TRIM28 transcriptional co-repressor possess DNA-binding domains. Alterations of chromatin structure due to TRIM28 recruitment to the specific sites of the genome using artificial construct lead to transcriptional repression of RNA polymerase I, II, and III promoters [35–37].

TRIM28 is bound to several different categories of binding sites within the genome, including promoters of non-ZNF genes and promoters and 3′ exons of ZNF genes. However, Iyengar S. et al. [38] demonstrated that the expression of most genes that are near TRIM28 binding sites was not regulated by TRIM28. Interestingly, the effects of loss of TRIM28 on the human transcriptome were very small, suggesting that the main role of TRIM28 may not lie in transcriptional co-repression through KRAB-ZNF/TRIM28 mechanism [38]. Moreover, recently published data suggest that TRIM28 recruitment by KRAB domain proteins is not always sufficient to warrant transcriptional repressive activity. Some KRAB-ZNF proteins are poor transcriptional repressors despite their ability to recruit TRIM28, while others show strong KRAB-dependent transcriptional repression, but no TRIM28 binding [39].

Recently, a novel role for the TRIM28 factor in the control of transcriptional elongation was identified [40, 41]. TRIM28 stabilizes the pausing of RNA polymerase II (Pol II) close to the transcriptional start site (TSS) in many unactivated genes [40], permitting Pol II accumulation and readying genes for induction. The modulation of Pol II pausing depends on TRIM28 phosphorylation status. When phosphorylated at Ser824, TRIM28 facilitates Pol II release from the pausing, resulting in rapid transcription of target genes. Moreover, Bunch H. et al. [41] have demonstrated that TRIM28 regulates the transcription of a subset of long non-coding RNAs (lncRNAs) in mammalian cells by similar mechanism of Pol II pausing at their TSS. Mammalian genomes encode a large number of non-coding RNAs that greatly exceed mRNA genes [42, 43], therefore we could speculate the great role of TRIM28 in regulation of genome-wide transcription.

Together, previous reports suggest, that the engagement of TRIM28 in regulation of transcription is very significant however, the complexity of this engagement still remains to be elucidated.

## DNA damage response (DDR) pathway and TRIM28 phosphorylation

**Fig. 5:**
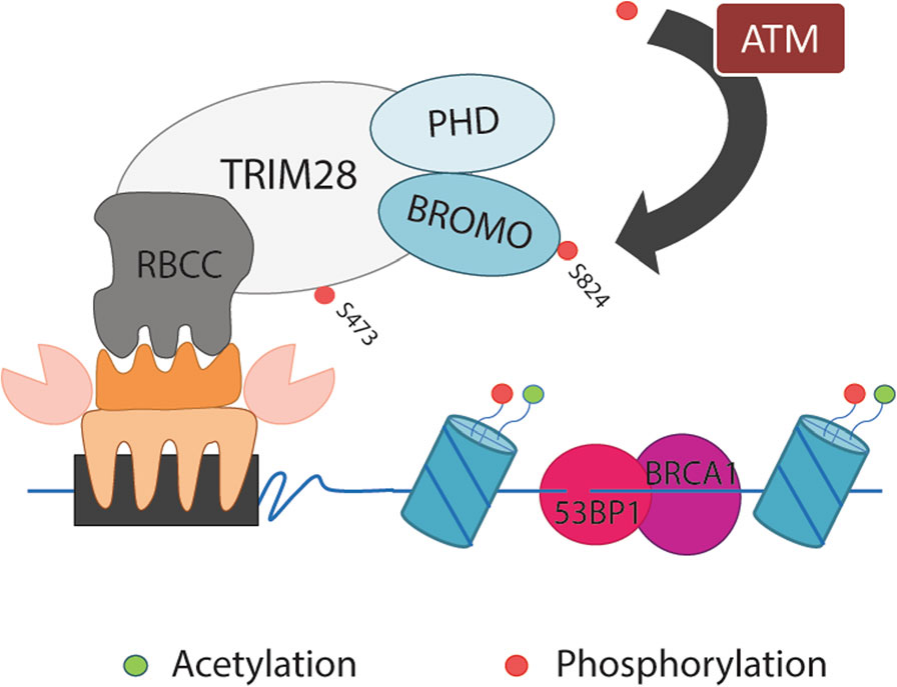
ATM-mediated phosphorylation of TRIM28 upon DNA double-strand break leads to destabilization of heterochromatin and enhances the accession of DNA repair machinery to the damaged site. In response to DNA damage, PI3-Kinase family members, like ATM, phosphorylate TRIM28 at serine 824 (near BROMO domain) and serine 473 (near HP1 binding domain). Serine 824 phosphorylation leads to abrogated NuRD and SETDB1 recruitment, and therefore halts heterochromatin compaction [47, 51]. Phosphorylation of TRIM28 at serine 473 attenuates its binding to the HP1 family proteins, and inhibits its transcriptional repression activity to KRAB-ZNFs target genes. Altogether, phosphorylation of TRIM28 results in chromatin relaxation that enhances the accession of DNA repair machinery (BRCA1 and 53BP1 proteins) to the damaged site [50]. BRCA1 – Breast Cancer 1; 53BP1 – p53 Binding Protein 1

The DNA damage response may be elicited in cell by many cytotoxic DNA lesions, such as double-strand breaks (DSB). This type of damage requires activation of Ataxia-Telangiectasia Mutated (ATM) kinase, a member of nuclear phosphatidylinositol-3 kinase–like (PIKK) family [44, 45]. Together with ATR (ATM and Rad-3 related) and DNA-PK (DNA-dependent protein kinase), ATM kinase triggers damage response pathway by phosphorylating several specific substrates, including H2AX histone, topoisomerase II binding protein 1 (TopBP1), p53 binding protein 1 (53BP1), and breast cancer 1 early onset (BRCA1), as well as itself. In the regions where nucleosome flexibility is constrained by heterochromatic factors (TRIM28, HP1, HDACs, etc.), repair proteins are unable to adequately access or manipulate the DSB [46]. Thus, ATM kinase phosphorylates TRIM28 protein on Ser824 within C-terminus and Ser473 near the HP1 binding domain, disrupting the interaction between TRIM28 and chromatin remodeling factors (Fig. 5). Diminished TRIM28 interactions with heterochromatin provide sufficient elasticity to facilitate DNA repair [47–51].

TRIM28 phosphorylation is a very early event of DNA damage response. White D. et al. [51] have shown that within 5 min after irradiation (9 Gy dose) the rapid, vigorous phosphorylation of TRIM28 protein occurred which decreased dramatically within 3 h after induction of DNA damage. Ser824-phosphorylation of TRIM28 is observed exclusively at the damage sites, from which TRIM28-Ser824-phospho spreads rapidly throughout the chromatin [48, 51, 52]. Ablation of the Ser824 phosphorylation site of TRIM28 leads to loss of chromatin decondensation induced by double-strand breaks within DNA and renders the cells hypersensitive to DSB-inducing agents [48]. Knocking down TRIM28, or mimicking a constitutive Ser824 phosphorylation of this protein (TRIM28-S824D mutant), leads to constitutive chromatin relaxation. Results obtained by Ziv Y. et al. [48] suggest that chromatin relaxation is a fundamental pathway in the DNA-damage response and identify its primary mediators. Mechanistic explanation of phospho-TRIM28-mediated heterochromatin relaxation in response to DNA damage-inducing agents was further demonstrated by Goodarzi A. et al. [49].

Interestingly, the mechanism of TRIM28-Ser824-phosphorylation-dependent regulation of chromatin relaxation was utilized by cancer cells to promote their growth. As presented by Bhatia N. et al. [53], in melanoma cancer cells MAGE-C2 (Melanoma-associated antigen-encoding gene) protein can induce ATM-dependent TRIM28-Ser824 phosphorylation favoring DNA damage repair mechanism over apoptosis. MAGE-C2 increases co-precipitation of TRIM28 with ATM and binding of MAGE-C2 to TRIM28 is necessary for increased TRIM28-Ser824 phosphorylation, resulting in enhanced DNA damage repair and, consequently, in promoting tumor progression [53].

TRIM28 Ser473 phosphorylation is also involved in efficient DNA repair and cell survival upon DNA damage [51, 54–56]. Depending on the type of DNA damage that occurs, TRIM28 Ser473 is phosphorylated by different DNA damage signaling pathways - Chk2 is the major kinase responsible for TRIM28 Ser473 phosphorylation in the etoposide- or IR-induced stress response, whereas Chk1 is required for TRIM28 Ser473 phosphorylation in response to UV radiation [55, 57–59]. However, unlike the phosphorylation at Ser824, the TRIM28 Ser473 phosphorylation is diffusely localized in the nucleus instead of accumulating at damage sites and forming foci [48, 51, 58]. Moreover, TRIM28 Ser473 phosphorylation proceeds in a slower kinetic manner compared with TRIM28 Ser824 phoshporylation. In their work, Hu C. et al. [57] have reported that DNA damage-induced TRIM28 Ser473 phosphorylation attenuates its binding to HP1 family proteins, which may lead to an increased binding of TRIM28-E2F1 and reduce the ability of E2F1 to activate the expression of a subset of proapoptotic genes and apoptosis. Therefore, TRIM28 contributes to the negative regulation of E2F1 and may serve as a partial backup to prevent E2F1-mediated apoptosis in cancer cells [57, 60], suggesting that TRIM28 acts as a pro-tumorigenic factor.

## Regulation of epithelial-to-Mesenchymal transition (EMT)

The Epithelial to Mesenchymal Transition (EMT) is defined by the loss of epithelial characteristics, mainly cell polarity and cell-cell contacts, and the acquisition of a mesenchymal phenotype [61–63]. EMT may be triggered by multiple extracellular stimuli and transcriptional regulators, but how such distinct signaling pathways orchestrate the complex cellular events that facilitate EMT is not well understood yet [63–65]. EMT was initially recognized during several critical stages of embryonic development and has been implicated in promoting carcinoma invasion and metastasis [63, 64, 66]. Hallmarks of EMT include: (i) the decreased expression of cell adhesion molecules such as E-CADHERIN [63]; (ii) the upregulation of MMPs to assist in the degradation of the basement membrane [67]; (iii) the activation of the Rho/Cdc42 family small GTPase which are necessary to cytoskeleton rearrangement [68]; and (iv) the nuclear translocation of several transcription factors such as β-CATENIN, TCF/LEF1 (T Cell Factor/Lymphocyte Enhancer Factor 1) complex, SNAI1, SNAI2 (also known as SLUG), SIP-1 and TWIST1 [69].

**Fig. 6:**
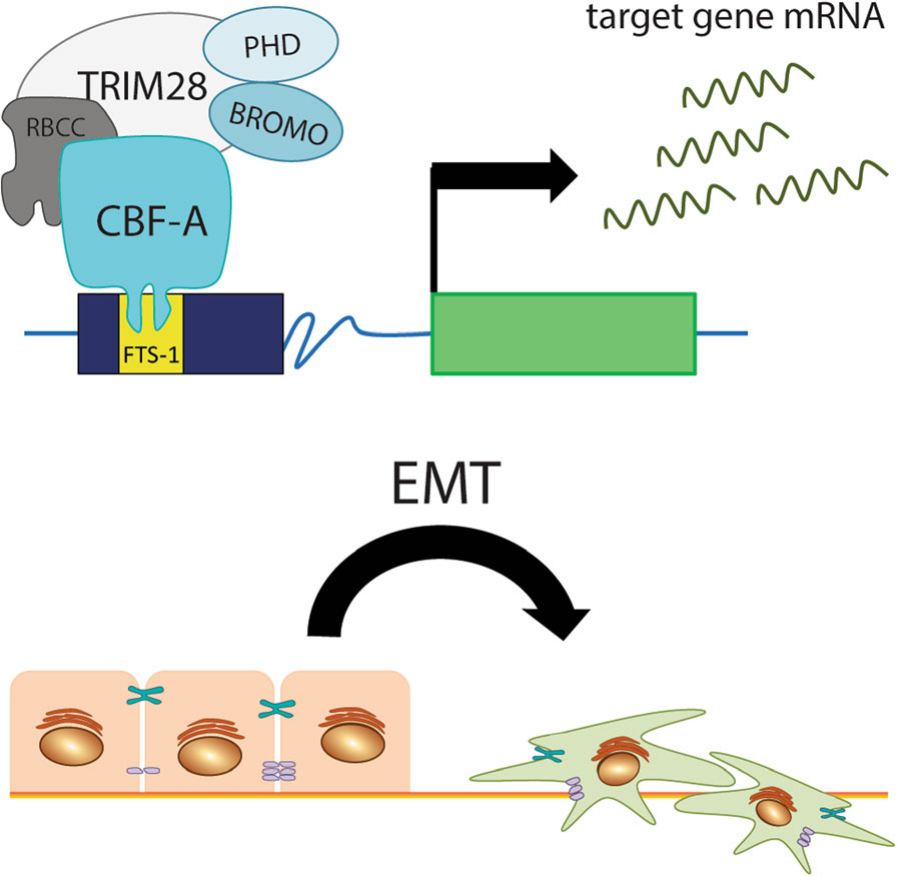
TRIM28 together with CBF-A bound to FTS-1 sequence forms a proximal activator of transcription in epithelial-mesenchymal transition, enhancing the expression of EMT transcriptome. CBF-A/TRIM28 complex binds to FTS-1 (fibroblast transcription site-1) response elements that exist in the promoters of genes modulating EMT transcriptome (including promoters of TWIST1, SNAI1, HMGA2, LEF1, ETS1, etc.) leading to acquisition of mesenchymal phenotype and loss of epithelial-related gene expression. Formation of CBF-A/TRIM28/FTS-1 is sufficient for the induction of epithelial-mesenchymal transition

Additionally, the expression of some of these EMT inductors has been detected in a variety of human cancer biopsies, including breast carcinomas [70], and their overexpression is usually related to increased tumor aggressiveness or recurrence, unfavorable clinicopathologic variables, and poor prognosis [62, 64, 66]. Recently, a novel master regulator of EMT was described – a protein-DNA complex composed of TRIM28 protein, CBF-A (CArG box–Binding Factor–A) and the FTS-1 element (Fibroblast Transcription Site–1), the crucial element for the expression of FSP1 (calcium-binding Fibroblast-Specific Protein 1) in fibroblasts (Fig. 6) [71]. FTS-1 sites are present in the promoter regions of multiple genes involved in the EMT process [72], and the CBF-A and TRIM28 proteins recognize and bind to the FTS-1 sites in the genomic DNA controlling the expression of a wide spectrum of EMT responsive genes. Occupancy of the FTS-1 site by these proteins in the chromatin of epithelia transitioning to fibroblasts correlates with the activation of the EMT proteome [71].

Recently, Lin LF. et al. [16] demonstrated that TRIM28 can promote the invasion of cancer cells and that the acquisition of metastatic properties tends to be associated with EMT regulation. Similarly, Yu C. et al. [73] have observed that TRIM28 overexpression induced the EMT in pancreatic cancer cells both in vitro and in vivo, as indicated by increased expression of mesenchymal markers such as vimentin and decreased expression of E-CADHERIN, suggesting TRIM28 role in promoting metastasis. Furthermore, Chen L. et al. [74] demonstrated that TRIM28 plays a role in TGF-β-induced EMT in non-small cell lung cancer cells. TRIM28 expression is induced following TGF-β treatment, leading to enhanced cell migration and invasion in vitro. This effect is impaired with TRIM28-specific siRNAs. Moreover, TRIM28 has been demonstrated to regulate EMT gene expression (*CDH1* encoding E-CADHERIN and *CDH2* encoding N-CADHERIN) through modification of histones of target gene promoters, further implying its role in acquisition of highly aggressive mesenchymal phenotype of cancer cells [74]. Recently, Wei C. et al. [26] have reported that TRIM28 promotes breast cancer metastasis by stabilizing TWIST1 protein – transcription factor considered to be a master regulator of EMT. Overexpression and depletion of *TRIM28* led to upregulation and downregulation of TWIST1 protein, but not the mRNA levels of *TWIST1*, respectively. Knockdown of *TRIM28* resulted in TWIST1 downregulation with concurrent upregulation of E-CADHERIN and downregulation of N-CADHERIN that consequently inhibited breast cancer cell migration and invasion in vitro [26]. Therefore, TRIM28 regulates specific mediators of EMT at both transcriptional and posttranscriptional levels.

Importantly, EMT is relevant to the acquisition and maintenance of stem cell-like characteristics and is sufficient to endow differentiated normal and cancer cells with stem cell properties [75, 76]. Moreover, CSCs often exhibit EMT properties. This reciprocal relationship between EMT and CSCs might have many implications in tumor progression and previously demonstrated involvement of TRIM28 protein in regulation of EMT implies its role in cancer stem cell maintenance.

Therefore, TRIM28 is the first potential epigenetic regulator that promotes cell metastasis and may serve as a therapeutic target against cancer in combination with other traditional strategies [16, 77].

## Inhibition of p53 activity

The p53 protein plays a central role in tumor suppression maintaining genome stability and protecting cells against malignant transformation [78, 79]. In approximately half of the cancers, the *TP53* gene is deleted or harbors inactivating mutations. In remaining tumors that retain wild-type *TP53*, this gene is often inactivated via other genetic or epigenetic alterations [80]. The p53 is regulated by multiple signaling pathways and mechanisms, however, its activity is suppressed mainly by its major regulator - MDM2 protein [81–83]. MDM2 binds p53 and functions as an ubiquitin E3 ligase to promote p53 ubiquitination and degradation in the proteasomes [81–83]. MDM2 interacts with a variety of regulatory factors [84]. Among others, TRIM28 was identified as a MDM2-binding protein promoting p53 destabilization [77, 85].

**Fig. 7:**
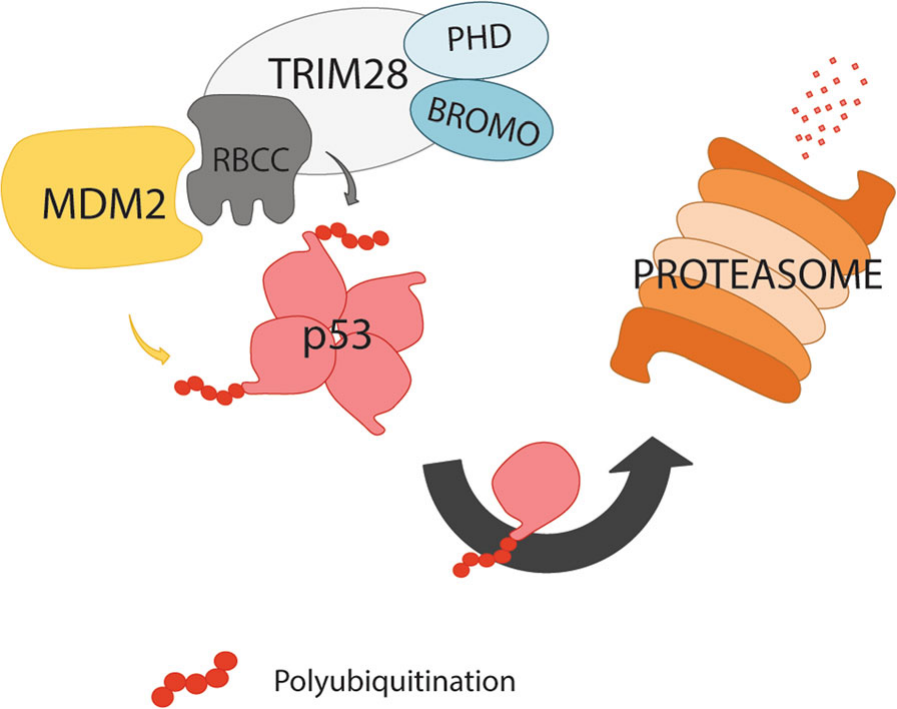
TRIM28 possesses an intrinsic E3 ubiquitin ligase activity (RING domain) and subsequently directs p53 for proteasomal degradation. TRIM28 directly interacts with MDM2 through its’ coiled-coil (CC) region within RBCC domain and cooperates with MDM2 to promote p53 polyubiquitination and degradation in the proteasome. MDM2 - E3 ubiquitin-protein ligase, major regulator of the p53 tumor suppressor; p53 – tumor suppressor, guardian of the genome, tetrameric in active conformation

TRIM28 interacts through its coiled-coil (CC) domain with the central acidic domain of MDM2 and this interaction may recruit TRIM28 C-terminal-associated cofactors, such as histone deacetylases. Together with MDM2, TRIM28 protein stimulates the formation of p53–HDAC1 complex and inhibits p53 acetylation. Because acetylation and ubiquitination of p53 use common lysine residues and are mutually exclusive events, the ability of MDM2 to recruit TRIM28 would cooperatively deacetylate and then ubiquitinate p53. Indeed, as shown by Wang C. et al. [85] the RING domain of TRIM28 protein possesses the activity of E3 ubiquitin ligase and enhances p53 ubiquitination level in an MDM2-dependent fashion (Fig. 7). Cells with reduced endogenous *TRIM28* level are highly sensitive to p53 activation and apoptosis after DNA damage. Moreover, *TRIM28* reduction markedly enhanced the induction of p21^Cip1/Waf1^, a product of the p53 target gene, after treatment with actinomycin D or γ-irradiation resulting in reduced entry of cells in S phase of cell cycle [77, 85].

Therefore, TRIM28 as a novel MDM2-binding protein contributes to p53 functional regulation leading to inhibition of p53 acetylation and subsequent proteasomal degradation [77, 85].

Moreover, the formation of TRIM28-MDM2-p53 complex is enhanced by class I MAGE proteins (MAGE-A, MAGE-B and MAGE-C protein families), which are highly expressed in various cancers [79]. MAGE proteins together with TRIM28 actively promote tumor survival by facilitating MDM2-mediated suppression of p53 activity. However, further studies by Doyle JM. et al. [86] demonstrated that TRIM28 may ubiquitinate p53 independently from MDM2 protein. MAGE proteins enhance the ubiquitin ligase activity of RING domain proteins, such as TRIM28 protein, targeting p53 for degradation in a proteasome-dependent manner independently from MDM2 ubiquitin ligase. These results are consistent with TRIM28 tumorigenic functions.

## Maintenance of stem-cell characteristics

Embryonic stem cells (ESCs) have been used extensively as a model system to study early mammalian development and the molecular control of self-renewal and pluripotency [87, 88]. For the first time, the role of Trim28 protein in embryonal development in mice was determined in 2000 by Cammas F. et al. [15]. Homozygous *Trim28* ^*−/−*^ (null mutant) embryos, which developed normally until the blastocyst stage and underwent uterine implantation, were arrested in their development at about embryonic day (E) 5.5 (early egg-cylinder stage) and failed to gastrulate, indicating that Trim28 is essential for early postimplantation mouse development [15]. As presented below, TRIM28 is essential for keeping cells in their intrinsic “state of cell differentiation”, facilitating both stem cell maintenance and inhibiting reprogramming of somatic cells.

In 2009 Hu G. et al. [89] have presented the genome-scale functional genetic screen in mouse ESCs for pluripotency genes, which detected Trim28 as a protein involved in regulation of the expression of pluripotency markers, such as Oct-4 (Octamer-binding Transcription Factor 4), Sox2 ((Sex determining region Y)-Box 2) and Nanog. In their work, Hu G. et al. [89] discovered that *Trim28* depletion resulted in significant down-regulation of *Oct-4*, *Sox2* and *Nanog* mRNA expression level and have led to differentiation of ESCs into the primitive ectoderm lineage. Together with other pluripotency markers, Cnot3, Zfx and c-Myc, TRIM28 co-occupies many putative gene promoters and forms a unique module in the self-renewal transcription network that is distinct from the module of Nanog–Sox2–Oct-4. Intriguingly, target genes of all four factors (Cnot3, Trim28, c-Myc, and Zfx) are enriched for cancer genes (as determined by Ingenuity Pathway Analysis) supporting the idea that regulatory networks controlling self-renewal in stem cells may also be active in certain cancers [89].

Furthermore, Seki Y. et al. [90] have demonstrated that phosphorylated at Ser824 Trim28 protein formed a complex with the pluripotency-specific transcriptional factor Oct-4 on the promoters of pluripotency-specific genes, and promotes not only the gene expression of pluripotency-specific transcriptional factors such as Nanog, Sox2, and Oct-4, but also the expression of various chromatin remodeling proteins for efficient control of pluripotent ES cell in a phosphorylation-dependent manner. Also, Jin VX. et al. [91] using ChIP-ChIP analysis combined with a unique bioinformatics approach demonstrated that half of the promoters bound by OCT-4 and SOX2 were co-occupied by TRIM28 in embryonic carcinoma cells. Moreover, Trim28 protein was recently demonstrated to be SUMOylated in mouse embryonic stem cells by Sumo2 in order to establish provirus silencing through the canonical Zfp809/Trim28/Eset machinery [92] which ultimately safeguards ESC genome integrity. Also, Cheng B. et al. [93] reported that in contrast to inducing the expression of pluripotency markers, Trim28 has opposite effects on differentiation-inducible markers in mouse ESC. Together with Polycomb repressor 1 (Prc1) complex, Trim28 is bound at the promoters of differentiation-inducible genes repressing the transcription, which further supports the requirement for Trim28 in the maintenance of mESC pluripotency.

Trim28 was also identified as an epigenetic barrier to induced pluripotent stem cell reprogramming [94, 95]. Knockdown of Trim28 during reprogramming of mouse embryonic fibroblasts resulted in increased expression of genes located in repressive chromatin regions (marked with H3K9me3 modifications) and upregulation of specific endogenous retroviruses, indicating a de-condensed and active chromatin state that facilitates the transition through reprogramming [94]. Therefore, Miles DC. et al. suggested that Trim28 safeguards the differentiated state of somatic cells by maintaining the repression of these regions. Interestingly, Klimczak M. et al. [95] reported that even if depletion of Trim28 increases the relaxation of chromatin and in consequence facilitates the reset of differentiated state, emerging iPS cells with downregulated Trim28 expression quickly lose their self-renewal potential and spontaneously differentiate demonstrating that Trim28 is indispensable for the maintenance of stable iPS cells.

Recently, the indispensable role of TRIM28 for maintaining breast cancer stem cell population has been reported [25, 96]. Cancer stem cells (CSCs) are rare, tumor-initiating cells that exhibit stem cell properties: capacity of self-renewal, pluripotency, highly tumorigenic potential, and resistance to therapy [97]. In our studies, we have demonstrated that downregulation of *TRIM28* in highly aggressive, undifferentiated cells of triple-negative breast cancer MDA-MB-231 cell line, which is highly enriched in vitro in CSC-like population characterized by CD44^+^CD24^−/low^ phenotype, led to significant (*p* < 0.0001) inhibition of tumor growth in vivo upon subcutaneous cell injection into athymic nude mice [25]. Global analysis of gene expression (RNA Seq and in silico analyses) of MDA-MB-231 wild-type and TRIM28-downregulated xenografts revealed substantial reduction of pluripotency markers expression as well as significant inhibition of signaling pathways previously reported to control the complex mechanism of stem cell maintenance. Moreover, the loss of CSC population in TRIM28-depleted MDA-MB-231 xenografts was further validated with limiting dilution assay (LDA) in vivo. Also, Li J. et al. [96] have observed that TRIM28 together with EZH2, a member of Polycomb Repressor 2 (PRC2) Complex, co-regulates a set of genes associated with stem cell maintenance and poor survival of breast cancer patients. *TRIM28* depletion in MCF7 breast cancer cell line in vitro resulted in significant inhibition of CD44^+^CD24^−/low^ mammosphere formation, which correlated with decreased expression of stem-cell associated genes. These results demonstrate substantial role for TRIM28 in activation of gene expression that promotes mammary stem cell enrichment and maintenance and further underlines TRIM28 engagement in promoting cancer progression [96].

Altogether, TRIM28 maintains both normal and cancer stem cells in the pluripotent state at least partially by repressing the genes associated with differentiation and inducing expression of stemness markers [25, 89, 90, 93, 96]. On the other hand, TRIM28 is an epigenetic barrier to induced reprogramming and downregulation of TRIM28 level facilitates rapid acquisition of stem-like phenotype upon exogenous expression of Yamanaka’s reprogramming factors [95, 96]. This collectively suggests that TRIM28 safeguards the intrinsic “state of cell differentiation”, maintaining stem cells and somatic cells in the pluripotent and differentiated state, respectively, and suggests that modulation of TRIM28 level may contribute to destabilization of cell differentiation state.

## Induction of autophagosome formation and role in the regulation of cancer cell metabolism

Cancer cells utilize various strategies like high glycolytic flux, redox signaling, and modulation of autophagy to avoid cell death and overcome nutritional deficiency. Autophagy allows the cancer cell to recycle intracellular proteins and organelles in lysosomes to provide an alternative source of energy during periods of metabolic stress [98, 99]. Recent reports demonstrated that autophagy is mechanistically linked to the maintenance of cancer stem cells [100–102] and enables these cells to overcome drug toxicity [103]. Interestingly, TRIM28 protein is involved in regulation of autophagy at several distinct levels [104–106].

**Fig. 8:**
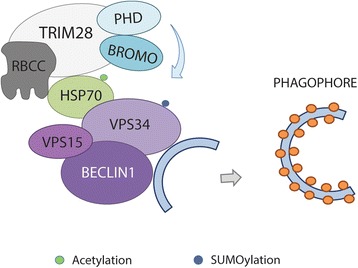
TRIM28-PHD-mediated SUMOylation of VPS34 protein in response to stress conditions stimulates the phagophore formation. Acetylated HSP70 recruits TRIM28 E3 ligase for SUMOylation of VPS34 at Lys840 increasing VPS34 activity bound to BECLIN1. Consequently, this complex promotes phagophore formation

Significant role for TRIM28 protein in coordination of phagophore formation was reported by Yang Y. et al. [104]. The induction and nucleation of the phagophore depends on the activity of the adenosine monophosphate (AMP)-activated protein kinase (AMPK) and mammalian Target Of Rapamycin Complex 1 (mTORC1)-regulated serine threonine kinase Unc-51–Like Kinase 1/2 (ULK1/2) complex [99, 107]. However, the phagophore formation also critically depends on the production and availability of phosphatidylinositol 3-phosphate [PI(3)P] which is controlled by the activity of VPS34 protein – Vacuolar Sorting Protein 34, the class III Phosphatidyl Inositol-3-OH Kinase (PI3K), also known as PI3K catalytic subunit type 3 (PI3KC3). VPS34 recruits the other autophagy regulatory proteins involved in phagophore and autophagic vesicle (AV) formation [107, 108]. Together with its binding partner - BECLIN1, VPS34 forms a complex, which is further bound by TRIM28 protein (Fig. 8). As a SUMO E3 ligase (through PHD domain), TRIM28 protein mediates Lys840 SUMOylation of VPS34, increasing its activity and enhancing autophagosome formation. TRIM28 protein is recruited to VPS34-BECLIN1 complex by acetylated HSP70 protein in response to stress stimuli, including stress caused by exposure to anticancer agents such as etoposide or doxorubicin, or exposure to heat shock, UV light, or reactive oxygen species (ROS), which are well known to induce autophagy [104]. Moreover, TRIM28 is also involved in regulation of mitophagy – a selective degradation of mitochondria by autophagy [109]. Together with KRAB-ZNF proteins, TRIM28 repress the expression of specific microRNA that target mitophagy-associated genes and therefore, sustain the proper mechanism of mitophagy during the erythropoiesis. However, it is not known whether similar mechanism is utilized by cancer cells.

On the other hand, TRIM28 protein was demonstrated in a complex with MAGE-A protein, forming MAGE-A-TRIM28 ubiquitin E3 ligase that targets a master cellular energy sensor and regulator AMPK kinase for ubiquitination and degradation, resulting in decreased AMPK signaling, amplification of mTORC signaling, and downregulation of autophagy [105, 106]. The authors suggest that expression of MAGE-A3/6 in cancer cells has the ability to act as a molecular switch to convert TRIM28 from a pro-autophagy (induction of phagophore formation) to an anti-autophagy factor by targeting AMPK for degradation [105, 106].

Moreover, formation of MAGE-TRIM28 ubiquitin ligase complexes was also demonstrated to promote the Warburg effect and hepatocellular carcinoma progression by targeting Fructose-1,6-biphosphatase (FBP1), a rate-limiting enzyme in gluconeogenesis, for degradation [110]. High expression of TRIM28 increased glucose consumption and lactate production by promoting FBP1 degradation in hepatocellular carcinoma and resulted in stimulation of cancer cells growth both in vitro and in mice model. These results strongly evidence TRIM28 pro-tumorigenic activity.

Clearly, TRIM28 involvement in the regulation of cancer cell metabolic state is complicated and requires further analyses.

## Repression of transposable elements

Genetic instability is one of the key features associated with development and progression of cancer. Among other factors contributing to this instability, transposable elements (TE) have been reported to cause several types of cancer through insertional mutagenesis of specific genes critical for suppressing or driving malignant transformation [111]. Although a large proportion (about 44%) of the human genome is occupied by transposons and transposon-like repetitive elements, only a small proportion (less than 0.05%) of these elements remain active today [112, 113]. Human TEs include members of both DNA and RNA families of transposons. The activity of RNA transposons, also termed retroelements, is controlled in embryonic stem cells by a rigorously orchestrated epigenetic process, engaging KRAB-ZNF repressors together with their cofactor Trim28 [114–116].

Ten years ago, Wolf D. and Goff SP. [117] have identified TRIM28 as a component of the repressor binding site (RBS)-binding complex, mediating epigenetic silencing of retroviruses in embryonic stem cells and embryonic carcinoma cells. Later, Rowe HM. et al. [114] have shown that Trim28 depletion resulted in upregulation of a range of retroelements, in particular intracisternal A-type particles (IAP), in mouse embryonic stem cells and in early embryos. Trim28 downregulation led to the loss of histone 3 lysine 9 trimethylation (H3K9me3) repressive chromatin mark localized at the 5′ untranslated region (5′UTR) of IAP genomes and therefore, to overexpression of IAPs [114]. Moreover, in Trim28-depleted mouse embryonic stem cells, repressive chromatin marks positioned at retroelements are replaced by histone modifications typical of active enhancers, stimulating transcription of nearby cellular genes, notably those harboring bivalent promoters [115]. Trim28-mediated control of retroelement expression is therefore crucial to safeguard the transcriptional dynamics of early embryos. In contrast, loss of Trim28 in mouse embryonic fibroblast (MEF) cells did not lead to an upregulation of retrotransposon expression, indicating a minor role for retrotransposon silencing in differentiated cells [114, 115, 118]. However, it was recently reported by Van Meter M. et al. [119] that Trim28 enforces the silencing of L1 (LINE 1 retroelement) in mouse fibroblasts by coordinating the packaging of these transposable elements into transcriptionally repressive heterochromatin upon mono-ADP ribosylation by SIRT6 protein. This modification is critical for the recruitment of HP1α and additional silencing factors at the target locus. However, they do not explain how Trim28 is recruited to specific site of the chromatin where the retroelement L1 is positioned. Castro-Diaz N. et al. [120] identified a novel KRAB-ZFP responsible for tethering Trim28 to and controlling the expression of a subset of L1 retroelements in mouse embryonic stem cells, strongly suggesting that these DNA-binding proteins are collectively involved in recognizing this class of retroelements [121]. Analogous results were further obtained in human embryonic stem cells [122].

However, it is still questionable whether similar mechanisms may be governed by cancer cells. In contrast to normal cells, the majority of human cancers, and cancer-derived cell lines, support variable, but typically much higher endogenous full-length L1 mRNA expression [111, 123, 124] despite frequent TRIM28 upregulation. Recently, Mita P. et al. [125] have reported significant role in regulation of L1 expression mediated by novel URI-PP2A-TRIM28 complex in prostate cancer cells. URI (Unconventional prefoldin RPB5 Interactor), a transcriptional repressor that interacts with RNA polymerase II [126], was shown to bind and induce de-phosphorylation of DNA-tethered TRIM28-Ser824 by recruitment of PP2A phosphatase, resulting in transcriptional repression of L1 retroelement. In their work, Mita P. et al. [125] have observed significant upregulation of L1 mRNA level upon TRIM28-Ser824 phosphorylation (and chromatin decondensation), which was elicited by depletion of URI and consequent inhibition of PP2A recruitment to TRIM28 phospho-Ser824. Therefore, retroelements usually kept dormant in heterochromatic regions are tightly controlled by TRIM28 phosphorylation status. It is worth to consider whether DNA damage inducing agents triggering misregulation of DNA structure through altered phosphorylation of TRIM28 may consequently result in aberrant regulation of retroelements in cancer cells.

## Conclusion

Role of TRIM28 in cancer cells has been questioned for more than 15 years. The multitude and complexity of TRIM28 actions in cancer make it difficult to unambiguously establish whether TRIM28 is a cancer promoting agent or possess anti-proliferative activity. Therefore, cancer-associated proteins that were previously reported to interact with TRIM28 or be post-translationally regulated by TRIM28 are summarized in Table 1. Number of reports suggest that TRIM28 may serve as a pro-tumorigenic factor that when highly expressed, may facilitate cancer progression and metastasis by inducing EMT [71, 73, 74], mediating metabolic switch in stressed conditions [104–106] and downregulating the activity of p53 [77, 85, 127], which collectively enable cancer cells to progress through the cell cycle despite number of errors within the genome. In unstressed cancer cells TRIM28 represses the expression of pro-apoptotic genes: *TP53AIP1*, *BAX*, *BBC3* (PUMA) and *PMAIP1* (NOXA), further suggesting that TRIM28 provides survival advantage to cancer cells [128, 129].

**Table 1:** The cancer-associated TRIM28 targets or interacting proteins

Target or interacting protein	Modification	Effect	Outcome	Oncogenic or tumor suppressive	Ref.
p53	deacetylation/ poly-ubiquitination	inactivation/degradation of p53	suppression of p53-mediated apoptosis and promotion of tumor cell survival	oncogenic	[77, 85]
AMPK	poly-ubiquitination	degradation of AMPK	amplification of mTORC signaling, downregulation of autophagy	oncogenic	[105, 106]
FBP1	poly-ubiquitination	degradation of FBP1	stimulation of the Warburg effect and cancer progression	oncogenic	[110]
VPS34 (PI3KC3)	SUMOylation	activation of VPS34	induction of phagophore formation	oncogenic	[104]
E2F1	HDAC1-mediated deacetylation	inactivation of E2F1	suppression of E2F1-mediated apoptosis after DNA damage in p53-null cells	oncogenic	[57, 60]
E2F3, E2F4	HDAC1-mediated deacetylation	inactivation of E2F3, E2F4	repression of members of the E2F family that are critical for cell proliferation	tumor suppressive	[5]
MAGE-C2	none	interaction with MAGE-C2 increases co-precipitation of TRIM28 with ATM upon genotoxic stress	enhanced repair of damaged DNA	oncogenic	[53]
CBF-A/FTS-1	none	interaction with CBF-A/FTS-1 induces the expression of VIM, FSP1 and other mesenchymal markers and represses the expression of epithelial markers (CDH1, ZO-1, etc.)	activation of EMT proteome	oncogenic	[71]
TWIST1	none	interaction and stabilization of TWIST1	promoting invasion and cell migration	oncogenic	[26]
URI-PP2A	none	de-phosphorylation of TRIM28 (at Ser824), chromatin condensation	repression of L1 retroelement	tumor suppressive	[125]

TRIM28 interaction with KRAB-ZNFs was omitted in this summary due to diverse and context-dependent role of these transcription factors [130]

However, there are thousands of TRIM28 binding sites within the genome but only few cellular genes that respond to loss of TRIM28 [38], which might suggest that a major role of TRIM28 is to generally guard genome integrity. Therefore, TRIM28 prevents genome destabilization and parallelly, blocks the path of cancer development. Also, tight regulation of retroelement repression suggests that TRIM28 acts as an anti-tumorigenic factor, safeguarding normal cell from cancer transformation in a phosphorylation-dependent manner [125]. However, phosphorylation of specific TRIM28 residues is also connected with DNA damage response pathway and TRIM28-Ser824-phosphorylation-dependent regulation of chromatin relaxation is utilized by melanoma cancer cells to promote their growth [53]. Furthermore, TRIM28 is an indispensable regulator of stem cell pluripotency, facilitating self-renewal of both normal and cancer stem cells [25, 90] and TRIM28-dependant maintenance of CSC population certainly represents pro-tumorigenic function of TRIM28.

Therefore, the mode of TRIM28 action is highly context-dependent, with many reports strongly implying its’ cancer promoting features. Undoubtedly, further studies are necessary to define the exact role of multi-domain TRIM28 protein in cancer development and progression. Also, better understanding of TRIM28 involvement in carcinogenesis may help to answer whether TRIM28 possess the potential to become a new anti-cancer target.
